# Hyperspectral imaging and healthy aging: an observational study using hand skin as surface for monitoring healthy aging processes

**DOI:** 10.1007/s10522-026-10461-w

**Published:** 2026-06-17

**Authors:** Maddalena M. Bolognesi, Teresa Sassetti, Martina Caramenti, Chiara Ceriani, Gloria Bertoli, Tecla Aramini, Marcella Bonanomi, Daniela Gaglio, Michele Caccia, Francesca Gallivanone

**Affiliations:** 1 Institute of Bioimaging and Complex Biological Systems, National Research Council, Segrate, Italy; 2 National Biodiversity Future Center, Palermo, Italy

**Keywords:** Hyperspectral imaging, Skin, Healthy aging, Exposome

## Abstract

With the global rise in life expectancy, promoting healthy aging has become a central focus in biomedical research. From global initiatives like The World Health Organization’s Decade of Healthy Ageing (2021–2030) to local ones, they highlight the need for accessible, non-invasive, and cost-effective tools to monitor aging-related physiological changes. Since the hand skin is an easily accessible tissue, it can offer valuable insights into aging processes, influenced by age, gender, and environment, as suggested by the paradigm of One Health. In this study, we exploited the potential of Visible and Near-Infrared Hyperspectral Reflectance Imaging (Vis-NIRHSI) as a quantitative, non-invasive method for monitoring skin aging in healthy individuals. A dataset of 101 hand-skin hypercubes images, from healthy volunteers over 40 years old, was acquired with a dedicated camera and analyzed in the range of 410 – 820 nm. A total of 224 spectral and texture features were extracted at selected wavelengths to investigate age-related variations in skin properties and to establish a standard reference map. Among these, 15 were retained as significant descriptors of the aging process and were used to build reference curves for the “healthy status condition.” A questionnaire about quality and perception of lifestyle was administered to describe the sampled population in terms of exposome. Our findings suggest that hyperspectral skin analysis can serve as a promising biomonitoring approach for assessing aging dynamics and may contribute to the development of reliable tools for supporting healthy aging monitoring strategies.

**Supplementary Information:**

The online version contains supplementary material available at https://doi.org/10.1007/s10522-026-10461-w.

## Introduction

Aging is a complex biological process, lacking a unique definition and consensus among researchers (Gladyshev et al. 2024). In the last three decades, many studies have concurred to define hallmarks of aging, particularly at cellular and molecular level (López-Otín et al. 2013).

Beyond its biological dimension, aging also represents a major demographic phenomenon with life expectancy continuing to rise. The reaching of a mean value of 81.7 years in Europe in 2024 (Eurostat 2025), places it between East Asian countries such as Hong Kong, Japan, and South Korea (>84 years) and regions of Sub-Saharan Africa (<54 years). In parallel with this global increase in longevity, the number of older individuals is projected to more than double by 2050, reaching 2.1 billions worldwide (World Health Organization 2020).

As the global population continues to age, great attention is being devoted to strategies aimed at reducing healthcare costs while improving quality of life. In this context, different measures are being developed to promote healthy aging, to monitor its progression and to minimize the cost. Global initiatives, such as the World Health Organization–sponsored United Nations Decade of Healthy Aging (2021–2030) (World Health Organization 2020), highlight the urgent need for effective, scalable, and preventive approaches. Alongside these efforts, the identification of non-invasive tools capable of monitoring the aging population represents a key research objective.

Being one of the most visible and accessible indicators of age-related changes, the skin surface offers a unique opportunity to be easily and non-invasively monitored, reflecting a wide range of underlying biological processes, including variations in hydration, pigmentation, and vascularization. Evidence of this potential is provided by the rapid development of wearable technologies designed for continuous skin assessment. Wearable devices for real-time skin health monitoring represent emerging tools for personalized medicine, enabling the dynamic tracking of physiological parameters and supporting individualized preventive and therapeutic strategies (Frasier et al. 2024).

In fact, many factors affect skin aging, and they are typically divided into intrinsic and extrinsic processes (Venkatesh et al. 2019; Durai et al. 2012). The first group includes skin thickness, laxity, and wrinkles, while environmental factors (in particular UV exposure) and lifestyle belong to the latter.

In addition, other variables contribute to skin aging. Skin properties such as thickness, water content and melanin content, vary according to ethnic background, as well as gender (Durai et al. 2012). Due to these differences, a specific photoaging scale was developed for Caucasian skin, to specifically assess the severity of photodamage; however, its applicability is limited to this population (Glogau 1996; Oesch et al. 2022).

Taking into account gender differences, it is also known that hormones in women have effects on the skin. Estrogen, for example, is involved in wound healing and collagen synthesis, and as consequence, postmenopausal skin reflects these issues, by exhibiting skin dryness, with decreased elasticity (Venkatesh et al. 2019).

For all these reasons, skin surface may represent a proxy and a reliable indicator of the aging process and could provide a convenient and accessible means for assessing aging condition and overall health status.

In this study, we propose a non-invasive approach based on versatile technology such as Visible–Near Infrared Hyperspectral Reflectance Imaging (Vis–NIR HSI) to map the aging process in a healthy population. Usually, HSI captures the spectral response of the skin surface, in terms of reflectance, across the 400–1000 nm region of the electromagnetic spectrum. The acquired data (called hypercubes) are 3D cubes made by a series of images (called bands), whose features and properties were used in this observational study as a tool to monitor the skin aging status of healthy volunteers. This technology has already shown great potential for medical applications (Lu and Fei 2014), particularly for the optical evaluation of tissues samples and at different scales of magnitude. Biological tissues, in fact, modify their optical behavior, such as absorption, reflectance and scattering, in response to physiological and pathological processes (Cooksey and Tsai 2015). These changes provide valuable information, for example, in disease onset and progression. For these reasons, HSI imaging technologies have already been widely applied in the assessment of various medical conditions, including different types of cancer and their histological characterization (He et al. 2025; Wang et al. 2026; Zhang et al. 2026), skin lesions (e.g., cellulitis, psoriatic conditions and acne) (Chen et al. 2018; Odrzywołek et al. 2022; Zheng et al. 2025), necrosis (Atmodiwirjo and Rininta 2026), surgical pathology (Jong et al. 2025), and in the cosmetic field (Blaksley et al. 2022).

From hyperspectral data analysis, multiple optical parameters can be derived, enabling a comprehensive and non-invasive characterization of skin tissue. The most relevant are represented by the indirected measure of water content, Oxy-Hemoglobin (HbO) and Deoxy-Hemoglobin (HbR), and melanin presence (Zheng et al. 2025; Atmodiwirjo and Rininta 2026; Bjorgan et al. 2014).

Furthermore, additional estimations of tissue properties can be also derived by texture analysis of skin images, through radiomic features extraction (i.e. quantitative metrics usually extracted from medical scans like computed tomography, magnetic resonance imaging, or positron electron tomography, obtained using advanced computational algorithms) which allows the evaluation of variations in skin texture at finer level (Calin et al. 2016).

Combinations of these approaches have already been used for skin classification (Hoxha et al. 2025) and for the indirect measure of water loss dehydration (Hooper et al. 2014; Beck et al. 2021), tissue oxygenation (Linek et al. 2026), hemoglobin (Jorgensen et al. 2019; Liang et al. 2021) and texture modification, well known hallmark of aging, particularly detectable in skin.

This suggests that it is possible to obtain a referenced signature to assess the healthy aging process of the skin.

In the end, the non-invasive approach offered by hyperspectral imaging makes it useful and suitable for continuous or longitudinal monitoring without discomfort or risk to the subject. By combining the study of 2D features within hypercubes selected bands (spatial analysis) with the investigation of the spectra shape and characteristics as the wavelength varies (spectral analysis), HSI allows for a comprehensive tissue characterization and quantification of various optical parameters of the skin. Even if it exists a skin spectra databases (Lu et al. 2025a) and HSI has already been suggested as a suitable tool for assessing physiological changes related to aging (Calin et al. 2016), often the relationship between skin status and aging still relies on qualitative consideration (Durai et al. 2012). This work fits into this scenario and, identifying some parameters as effective for aging monitoring, it is aimed to fill these gaps and it moves a step forward toward the successful application of HSI to follow aging process.

## Data and methods

### Cohort and study design

This project was approved by the Ethics Committee of the National Research Council (CNR, Prot. N. 0180099 del 28/05/2024 UOP SI000002). A cohort of 101 healthy volunteers aged ≥ 40 years, residing in Lombardia and Piemonte (Northern Italy), Caucasian ethinicity, was recruited between June 2024 and March 2025, promoting the project via the website of the Institute of Bioimaging and Complex Biological Systems of the National Research Council (IBSBC-CNR) and via direct communication with associations of the area of Piemonte and Lombardia. Participants were invited to a dedicated study setting in IBSBC-CNR, where they underwent a series of non-invasive assessments and answered a questionnaire.

All participants provided written informed consent prior to enrollment. Inclusion criteria required age ≥ 40 and the absence of diagnosed diseases, while exclusion criteria included recent skin treatments and the presence of chronic conditions, with particular attention to skin-related diseases (e.g., psoriasis or other dermatological disorders), which could interfere with skin-based measurements. The Mini-Mental State Examination (MMSE) (Folstein et al. 1975) was administered to perform a preliminary check for possible cognitive impairments. Since MMSE score below 24 can be suggestive of cognitive decline, participants falling under this cutoff were excluded (Folstein et al. 1975).

Participants completed a questionnaire, allowing to obtain socio-demographic data as well as information about their lifestyle (see Supplemental Material). Questionnaire items were derived and adapted by the analysis of surveys proposed in the SHARE research infrastructure (https://share-eric.eu/data/data-access/citation-requirements). The questionnaire was implemented within the EUSurvey open source survey tool and administered on a personal computer to the participants during the experimental session. Adopting an a priori, top-down approach to the questionnaire, we defined a set of four coherent and conceptually meaningful domains, related to healthy aging: Perceived Well-Being, Protective Lifestyle Behaviors, Educational Level and Social Engagement (Supplemental Material). For each domain, a composite score was computed by considering the items belonging to it and used for subsequent analysis. In particular, domain’s scores were used to explore associations between exposome and skin-related measurements and to identify possible stratification or potential aggregation of data within the cohort. Descriptive statistics on scores were performed with respect to partecipants’ ages.

### Hyperspectral data

Hypespectral data acquisition was set within a dedicated room; to ensure illumination uniformity, four Led sources (19 W - 245 lm) were placed at the top of the acquisition set-up, $45^{\circ }$ oriented toward the sample (see Supplemental Fig. S1). Acclimatization time before image acquisition lasted approximately 15 minutes. All the data were acquired at room temperature (23 °C) and the volunteers have been asked to wash their hands with water before starting the experimental session. They were also asked not to use topical products on the day of acquisition. Volunteers hands were then laid on the bottom surface of the experimental set-up, 3 colored pins were positioned in order to help them to put their hands in the correct position under the camera (regardless the size of each person’s hand) and a member of the staff assisted them for assuring the correct position of the hand with respect to the pins. Data were obtained with a Specim IQ pushbroom camera (Specim Ltd., Oulu, Finland) placed above the sample at a distance of about 55 cm (corresponding to a resolution of 0.5 mm/pixel at the detector); the calibration process for the evaluation of the reflectance was repeated before each acquisition session by employing the dedicated white reference (99% reflectance) supplied by the manufacturer. The resulting data are hypercubes constituted by two spatial dimensions (images of 512 x 512 pixels) where each pixel is associated to a third dimension, the reflectance in the Vis-NIR range, constituted by 204 bands within 400 and 1000 nm (spectral resolution 7 nm) that accounts for the reflectance measured at that point of the surface of the volunteer’s hand. Hand hypercubes were acquired twice for each volunteer, one of the back and one of the palm. The dominant hand of the volunteer was chosen for data acquisition. A quality control check, based on visual inspection of the spectra, was performed at the end of each acquisition.

### Hyperspectral data analysis

The wavelength window used for the data analysis was set at 410 - 820 nm, since over 820 nm and under 410 nm the signal was observed to be impacted by white noise. However, for three spectral indices (Tissue Hemoglobin Index ($\textrm{THI}^{*}$), Tissue Water Index ($\textrm{TWI}^{*}$) and the Near Infrared Perfusion Index ($\textrm{NIRind}^{*}$) average values from spectral data were evaluated outside of this range. In the region >820 nm, the spectra appeared to be almost flat; the reflectance oscillates around an average value or, at least, shows a slight increase (or decrease). Therefore, averages over wavelengths windows large enough should provide a reliable values for parameters evaluation despite the white noise introduced by the detector. This hypothesis was also supported by the fact that the coefficient of variation (CV), defined as $CV_{R_{range}} = 100* (Std(R_{range})/ \langle R_{range} \rangle )$, was always less the 10% in the ranges that appear in the formulas defining the parameters.

For each hyperspectral acquisition, a ROI (Region of interest) was defined. Image segmentation was performed on a single spectral band (central within the 410 – 820 nm range) using 3D Slicer (van Griethuysen et al. 2017; Fedorov et al. 2012). A semi-automatic region-growing approach was adopted (Liang et al. 2021). The procedure was initialized by manually defining two seed regions (hand and background), after which the algorithm generated an initial segmentation that was subsequently refined to include the main dorsal or palmar surface while excluding fingers and wrist. A smoothing filter was finally applied to regularize the ROI boundaries (see Supplemental Fig. S2). This segmentation strategy, consistent with recommended practices in radiomics (Zwanenburg et al. 2020) (Parmar et al. 2014) (Hatt et al. 2017) ensures a stable and reproducible extraction of GLMC features.

The sub-hypercube, resulting from masking the hypercube with the defined ROI, was analyzed for each subject to extract a group of descriptors of hand-image–related aging conditions in health status. These descriptors were obtained by two different and complementary approaches based on spectral and texture analysis.

Spectral-based approach relies on the analysis of the behavior of the mean spectra in the ROI. Signal from sub-hypercube was normalized for what concerns reflectance and the mean spectrum of ROI was calculated. All spectra were plotted together to explore common behaviors. After this evaluation, for each spectrum, a selection of 16 putative significant parameters was extracted (Table 1). Wavelengths used to generate them correspond to known biological processes.

In detail the 16 parameters were so distributed: 4 parameters, called sigmoid parameters, were related to the typical growth that human skin reflectance displays between 575 and 760 nm and they were obtained by fitting the reflectance increase in this wavelength range according to a sigmoidal model (Supplemental Fig. S3); 8 parameters were selected according to literature, being connected to relevant biological processes such as the presence of melanin (rapidly decreasing absorption after 650 nm), the level of HbO and HbR (absorption bands at 650 and 800 nm, commonly used for tissue oxygenation), water content (wavelengths below 880 nm include the water absorption bands and can be seen as a measurement of the skin water content) and Erythema indices (560 and 650 nm); 4 parameters, called shape parameters, were derived from 4 notable points, obtained on the average spectra of each of the enrolled volunteers: i.e. the depths and heights of the two main reflectance maxima and minima identified respectively at 470 and 540 nm, and at 565 and 575 nm. The 16 parameters were assessed using custom scripts developed in Matlab (The MathWorks Inc 2024).

A radiomic approach was applied performing texture analysis on images at four fixed wavelengths and, in particular, where spectral analysis found the two main reflectance maxima and minima of mean spectra common to all enrolled volunteers (at 470, 540, 565 and 575 nm). Features extraction was performed using PyRadiomics Python package within 3D Slicer (van Griethuysen et al. 2017).

Data (2D ROIs selected) on the back and palm of each hand were imported into 3D Slicer software, where 24 gray-level co-occurrence matrix (GLCMs) texture features (Zwanenburg et al. 2020) for all the four considered wavelengths were extracted. The GLCMs were computed after image discretization in 64-gray levels and second order radiomic features, representing the relationships between the reflectance intensity of pixel pairs in image space at fixed distances, were extracted, depicting the texture of the sample surface (Ardakani et al. 2021).

Considering both spectral and radiomic analysis, for each volunteer, a total of 224 parameters were obtained: 16 from the mean spectral analysis and 96 from the radiomic approach applied to 4 waveleghts images for both the palm and back of the hand. HSI image parameters were joined to scores calculated from the questionnaire.

### Statistical analysis

Statistical analysis was performed in R (version 4.5.2) (R Core Team 2024). Univariate analysis was conducted on all HSI parameters by applying Kruskal-Wallis test with Bonferroni correction. Only those variables showing statistically significant differences in data stratification, according to the threshold defined on the base of the World Health Organization (WHO) recommendation (60 years), were retained for further analysis.

On the resulting significant variables, to avoid their redundancy in further analysis, features selection was performed. More in details relevant parameters were organized in different dataset: those from radiomic analysis according to their wavelenght of reference (four groups), those from spectral analysis into a unique one. For each of them was generated a correlation matrix, to explore relationship among the variables. Then a threshold was applied to the generated correlation matrices and, once identified clusters of correlated parameters, only those with the highest variance within each cluster were retained, to maintain the highest information for each groups of parameters (Gillies et al. 2016).

After features selection, the remaining variables were again evaluated individually by plotting an interpolated curve (spline) and their corresponding percentiles (p05, p25, p50, p75, p95) according to the decade of reference. Variables that showed no clear trend were discarded. Percentiles were plotted to determine the normal range of the sample, which consisted of healthy volunteer participants.

By combining the resulting non-redundant variables, a corresponding heatmap was generated, to evaluate samples distribution according to the selected parameters and to verify if they were eventually organized in cluster according to decades and questionnaire parameters.

After a global evaluation of the entire dataset, the same dataset was further stratified according to gender, and a betweeen-gender comparison was performed.

**Table 1:** List of spectral parameters extracted from the mean spectra, with the corresponding formulas and literature references. (*) indicates those indices adapted from (Pachyn et al. 2024). See Supplemental Material for a detailed explanation of the formulas

Type	#	Parameter	Formula	References
Sigmoid	1	Lower point ($y_L$)	$\displaystyle y = y_L + \frac{y_U - y_L}{1 + e^{-k(\lambda - \lambda _0)}}$	(Mancuso et al. 2024)
Sigmoid	2	Upper point ($y_U$)	$\displaystyle y_U = \text {Upper point}$	(Mancuso et al. 2024)
Sigmoid	3	Steepness (*k*)	$\displaystyle k = \text {Steepness}$	(Mancuso et al. 2024)
Sigmoid	4	Flex point ($\lambda _0$)	$\displaystyle \lambda _0 = \text {Flex point}$	(Mancuso et al. 2024)
Literature	5	Melanin Index (MI)	$\displaystyle 100 \log _{10}\!\left( \frac{1}{R_{650}}\right)$	(Silva et al. 2020)
Literature	6	Tissue Hemoglobin Index (THI$^{*}$)	$\displaystyle \frac{\frac{\mathrm {\langle R_{785}:R_{825} \rangle }}{\mathrm {\langle R_{530}:R_{590}\rangle }}-s_1}{s_2-s_1}$	(Pachyn et al. 2024)
Literature	7	Erythema Index ($EI_{1}$)	$\displaystyle 1000 \left[ \log _{10}\!\left( \frac{1}{R_{560}}\right) - \log _{10}\!\left( \frac{1}{R_{655}}\right) \right]$	(Stamatas et al. 2004)
Literature	8	Erythema Index ($EI_{2}$)	$$\begin{gathered} 100\left[ {\log _{{10}} \left( {\frac{1}{{R_{{560}} }}} \right) + 1.5\left( {\log _{{10}} \left( {\frac{1}{{R_{{540}} }}} \right) + \log _{{10}} \left( {\frac{1}{{R_{{580}} }}} \right)} \right)} \right. \hfill \\ \left. { - 2\left( {\log _{{10}} \left( {\frac{1}{{R_{{510}} }}} \right) + \log _{{10}} \left( {\frac{1}{{R_{{610}} }}} \right)} \right)} \right] \hfill \\ \end{gathered}$$	(Mancuso et al. 2024); (Saija 2000)
Literature	9	Tissue Oxygenation Index (TOI)	$\displaystyle \frac{R_{800} - R_{660}}{R_{800} + R_{660}}$	(Blackburn 1998)
Literature	10	Tissue Water Index (TWI^*^)	$\displaystyle \frac{\frac{\mathrm {\langle R_{955}:R_{980} \rangle }}{\mathrm {\langle R_{880}:R_{900}\rangle }}-s_1}{s_2-s_1}$	(Pachyn et al. 2024)
Literature	11	Normalized Difference Index (NDI)	$\displaystyle \frac{R_{800} - R_{650}}{R_{800} + R_{650}}$	(Rouse et al. 1974)
Literature	12	Near-Infrared Perfusion Index ($\textrm{NIRind} ^{*}$)	$\displaystyle \frac{\frac{\mathrm {\langle R_{825}:R_{925} \rangle }}{\mathrm {\langle R_{655}:R_{735}\rangle }}-s_1}{s_2-s_1}$	(Pachyn et al. 2024)
Shape	13	WS1	$\displaystyle \frac{R_{575} - R_{540}}{R_{575} + R_{540}}$	(Mancuso et al. 2024); (Sun et al. 2024)
Shape	14	WS2	$\displaystyle \frac{R_{560} - R_{540}}{R_{560} + R_{540}}$	(Mancuso et al. 2024); (Sun et al. 2024)
Shape	15	WS3	$\displaystyle \frac{R_{560} - R_{575}}{R_{560} + R_{575}}$	(Mancuso et al. 2024); (Sun et al. 2024)
Shape	16	WS4	$\displaystyle \frac{R_{575} - R_{478}}{R_{575} + R_{478}}$	(Mancuso et al. 2024); (Sun et al. 2024)

**Table 2:** Age distribution and MMSE-adjusted scores (MMSE$^{*}$) in the study cohort, shown overall and stratified by biological sex

	Age, Mean (s.d.), Median	40–49	50–59	60–69	70–79	>80	Total	MMSE$^{*}$ (s.d.)
Total	60.8 (11.1) 62	17	23	25	20	2	87	27.6 (1.7)
F	60.5 (10.8) 61	9	15	14	10	1	49	27.4 (1.6)
M	61.2 (11.6) 63	8	8	11	10	1	38	27.9 (1.7)

**Table 3:** Spectral and radiomic variables resulting from the feature selection analysis and list of those retained after redundancy evaluation based on the correlation matrix and variance

#	Variable	Selected
1	GLCM_Contrast_470_Palm	TRUE
2	GLCM_Idm_470_Back	TRUE
3	GLCM_JointEnergy_470_Back	TRUE
4	GLCM_ClusterProminence_470_Back	FALSE
5	GLCM_DifferenceEntropy_540_Back	TRUE
6	GLCM_MaximumProbability_540_Back	TRUE
7	GLCM_Contrast_565_Back	TRUE
8	GLCM_JointEnergy_565_Back	TRUE
9	GLCM_Contrast_575_Back	TRUE
10	GLCM_JointEnergy_575_Back	TRUE
11	Flex_Palm	TRUE
12	$\textrm{TWI}^{*}$_Back	TRUE
13	MI_Back	FALSE
14	Steep_Back	FALSE
15	NDI_Back	TRUE

## Results

### Cohort and study design

**Fig. 1:**
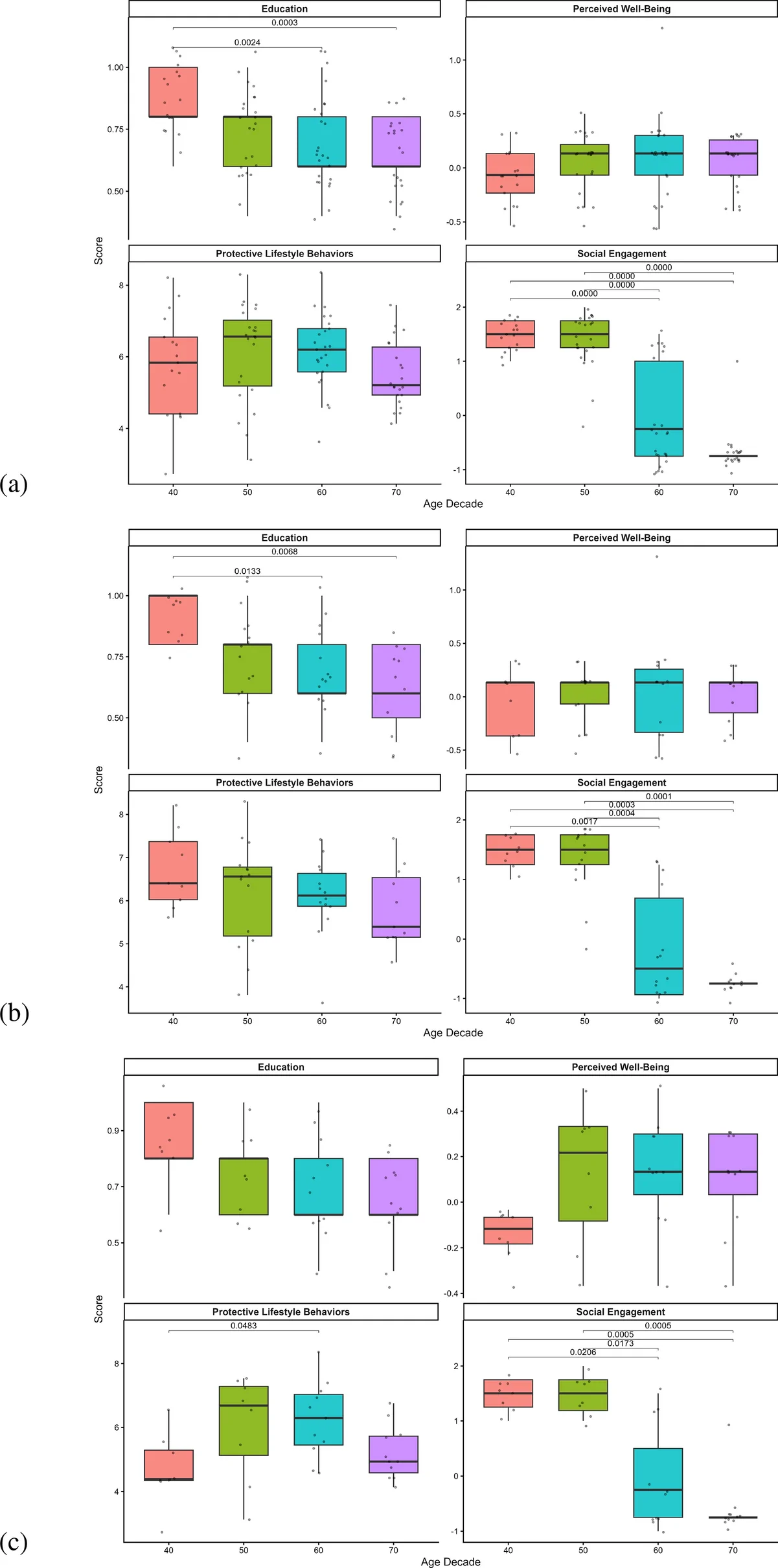
Box plots showing the distribution of questionnaire scores across decades. Participants older than 70 years were grouped together. Panel A shows the combined dataset, Panel B includes females only, and Panel C includes males only

101 volunteers participated in the study according to enrollment criteria. On the basis of MMSE scores adjusted for education level, 8 participants were excluded. Furthermore, image control excluded 6 volunteers for HSI poor image quality. The final data set was composed of 87 volunteers, uniformly distributed in terms of age (mean = 60.8 years, median = 62 years, sd = 11.1) and gender (F = 49, M = 38), and MMSE adjusted score (mean = 27.6, sd = 1.7) as reported in Table 2. Being the group ≥80 years underrepresented, it was then merged with the 70–79 decade for subsequent analysis. The individual evaluation of the four questionnaire scores across different age decades showed significant differences in two of the four examined variables (Fig. 1).

More specifically, Perceived Well-Being tends to increase modestly with age, while Social Engagement score exhibits a significant decline, especially comparing younger and older groups (Fig. 1a). Educational level shows a distinctive profile among the two groups: <60 and ≥ 60 years. Quality of life (Protective Lifestyle Behaviors) does not appear to have a clear trend, suggesting other influencing factors but not age. Similar trends emerge after stratifying the samples by gender (Fig. 1b,c), except for education in the male group, which confirms the global trend but not significantly and, on the contrary, shows a more important difference among 40–49 and 60–69 decades in Protective Lifestyle Behaviors.

Taken together, these results suggest the presence of two main age-based clusters, with a potential cutoff of around 60 years. This threshold is not surprising; in fact, according to WHO, from a socio-economical/demographic point of view, “older age” often begins around 60 years, which in many countries coincides with retirement and pension eligibility. Taking this into account, and on the base of WHO recommendation, 60 years was adopted as a reference threshold for all subsequent analysis.

### Hyperspectral data and statistical analysis

#### Mean spectrum

**Fig. 2:**
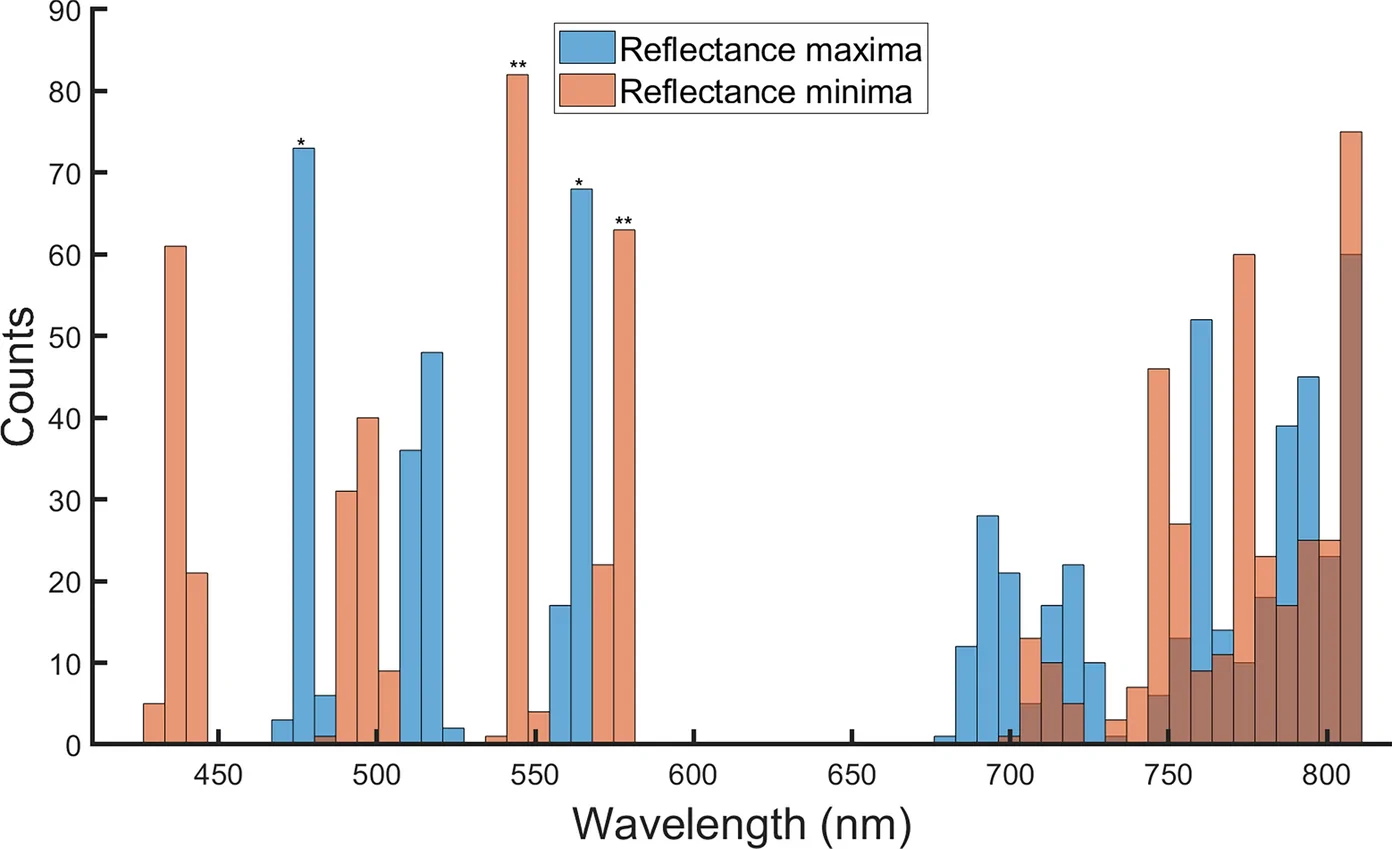
Histogram of notable points (min and max) of the mean spectra, * corresponds to 470 and 565 nm, ** correspond to 540 and 575 nm, wavlenghts selected for radiomic analysis. “Y” axis (Counts) reports the number of times a specific point has been detected at the corresponding wavelength

The mean spectra of all images plotted together showed great overlap, particularly at 470, 540, 565, and 575 nm, corresponding to main reflectance maxima and minima bands and indicating common and reproducible skin spectral features (Fig. 2).

**Fig. 3:**
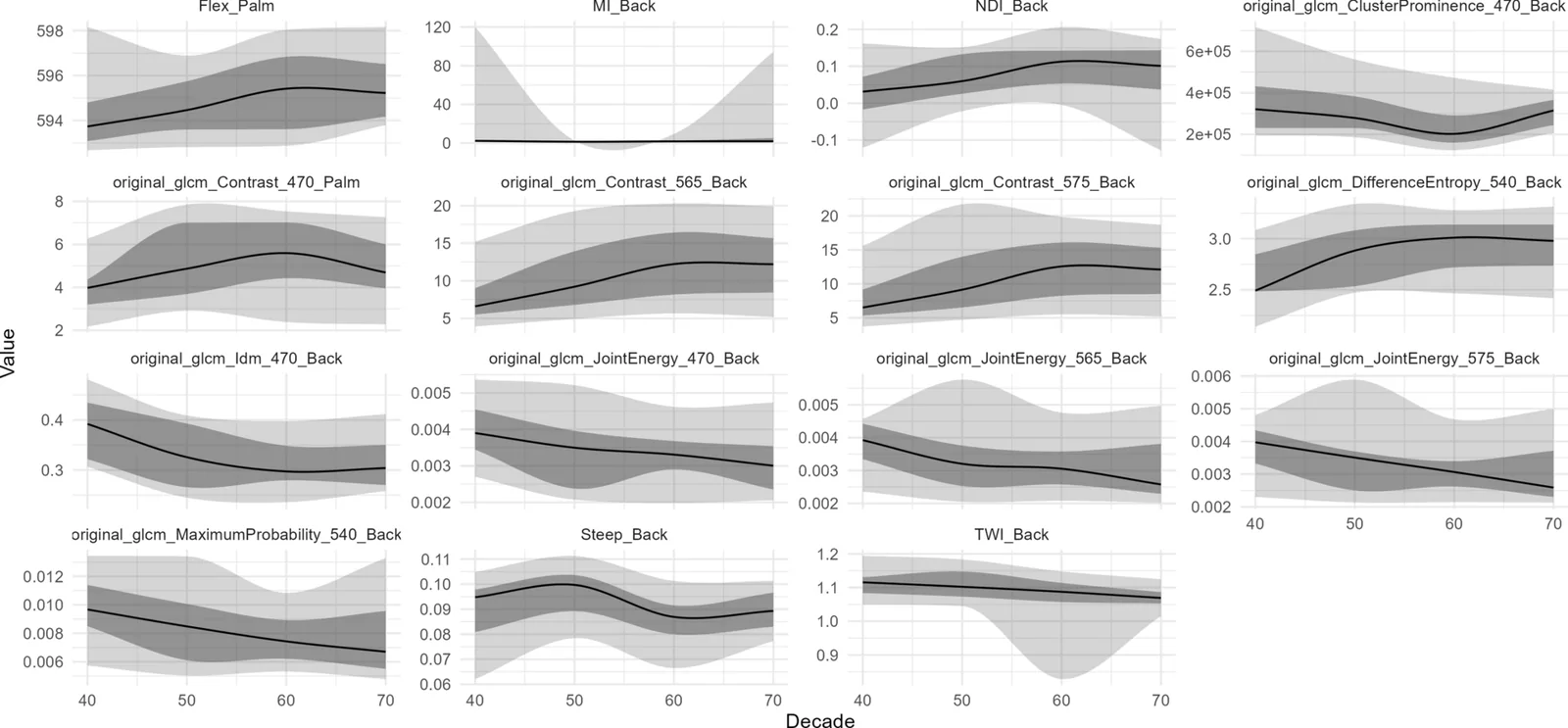
Reference curves of “healthy status” for the 15 parameters retained after feature selection plotted according to decades. Each curve is modeled using a spline function and corresponds to a single parameter. Outer light grey area corresponds to 5th–95th percentile range, inner dark grey area to 25th–75th percentile, line to median (50th percentile)

These values correspond to known parameters. 470 nm identifies the main skin characteristic absorption band in the wavelength range used for the evaluation of the carotenoid content. 540 and 575 nm correspond to the centers of the main absorption bands of HbO. 565 nm is the center of the reflectance maximum located at the middle of the W shape which characterizes human skin between 520 and 610 nm. These findings are reported for all kinds of skin (Sun et al. 2024) (Lu et al. 2025a) and were also verified on The Leeds Skin Database (Lu et al. 2025b) after selecting hand tissue of the Caucasian population aged ≥40 (data not shown). Therefore, these four wavelengths, were used to define the shape-derived parameters and to select images for extraction of radiomics features.

#### Univariate analysis

From univariate analysis 74/224 parameters were retained as significant if they were able to split the dataset into two distinct groups (≥ 60 and <60 years) on the base of WHO recommendation, belonging from both the spectral and radiomic analyses. After the grouping according to their wavelengh of reference (the radiomic features), and keeping the spectral into a unique one, they were evaluated in respect to their mutual relationships. The correspondent correlation matrices (Supplemental Fig. S4) confirmed the redundancy of many variables and guided the subsequent features selection. In particular, from the remaining 74 parameters, 15 parameters were retained by features selection and are reported in Table 3, divided as follows: five variables were derived from spectral parameters ("Flex_Palm", "MI_Back", "$\textrm{TWI}^{*}$_Back", "NDI_Back" and "Steep_Back"), and ten from texture analysis using GLCM features across different wavelengths: Contrast (470 nm Palm, 565 nm Back, 575 nm Back), Joint Energy (470 nm Back, 565 nm Back, 575 nm Back), Cluster Prominence (470 nm Back), Idm (470 nm Back), Difference Entropy (540 nm Back), and Maximum Probability (540 nm Back). In particular Contrast and Joint Energy were both reported three times per 470, 565, 575 nm, and these results are consistent with those reported by Calin and co-workers (Calin et al. 2016). Furthermore, dorsal side was preferred to palm, being selected on 13/15 features, probably because the back is prone to the greater exposure to external agents with respect to the palm (Calin et al. 2016). A reference curve (spline function) for each of the retained parameters was plotted to provide a range of "healthy status" according to age progression, as reported in Fig. 3 (and Supplemental Fig. S5). The vast majority of the parameters presents an ascending or descending trend, except for FlexPalm, Steep Back and MI Back, suggesting GLCM features being more reliable as descriptors of the process compared to Spectral values. An exception is represented by the $\textrm{TWI}^{*}_{Back}$ index and NDI_*Back*_ that behave like radiomic parameters. Based on these considerations, only 12 variables were considered suitable for multivariate analysis (indicated with TRUE in Table 3).

#### Multivariate analysis

**Fig. 4:**
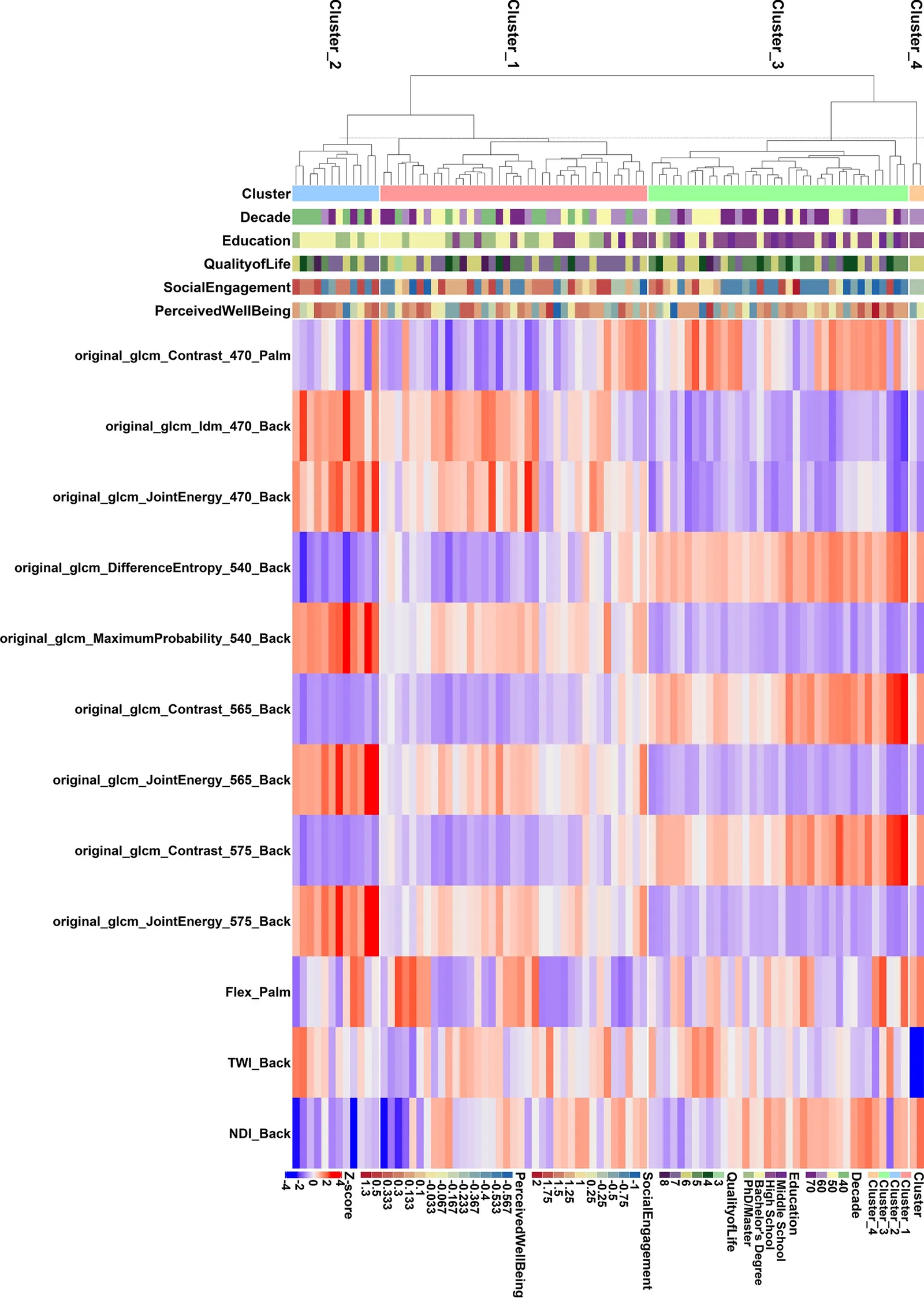
Heatmap derived from multivariate analysis with 12 final parameters, incorporating questionnaire score–related labels. The accompanying dendrogram on the top shows, with a cut at the four-cluster level, distinct groups named Cluster_1, Cluster_2, Cluster_3, Cluster_4

The above mentioned parameters were combined together to generate a descriptive heatmap (Fig. 4), aimed at investigate data relationships and eventually recapitulate correlations with questionnaire scores, age, and gender, by re-creating ordered groups. The analysis did not reveal any strong associations.

However, the hierarchical dendrogram accompanying the heatmap suggested the presence of three main groups (Cluster 4, presenting only two samples as represented in Fig. 4 was excluded). The first group (Cluster 2) seems to have a consistent signature of HSI parameters (the highest and the lowest) and is characterized by the presence of volunteers of the first/second decade, with high level education, high score of sociality, quite high Perceived WellBeing and variegated Quality of Life. The second group (Cluster 3) is composed by the older group, with a low level of education and social engagement but a high level of Perceived WellBeing. The third group, in the middle between them, is more heterogeneous.

Splitting the dataset according to gender (F/M), reflects this subdivision (data not shown).

## Discussion

Despite its controversial definition, aging is a progressive process which affects the whole organism during his life.

Globally, in this study, variables have been investigated between two groups, one constituted by the first two decades (40–59 years) and the second by volunteers (≥60 years), according to what suggested by the WHO, and results confirm this main subdvision. Questionnaire domains reflected it too, suggesting that volunteers over 60 years have mostly retired from work and usually live with one person (the spouse); consequently, they change their habits and often have a lower probability of interaction with other people. Also, the decline in educational score after the 50 s can be explained by the fact that the older volunteers had fewer opportunities for achieving a high scholar level.

Skin, as the most visible and exposed surface of the body, is the organ which better shows, in appearance and texture, the sign of aging progression.

HSI technology, thanks to its non-invasive manner and affordable cost, represents a viable and innovative tool for monitoring the aging process in healthy conditions. In this study, starting from a cohort of 101 volunteers, we characterized their hand skin profile to assess the range of variability while aging in healthy conditions. Many features have been extracted from ROIs selected sub-hypercubes, a group of them belonging to mean spectrum and a second one belonging to GLCM features.

Mean spectral shape obtained from data has been compared with International Skin Spectral Archive, after selecting the appropriate skin characteristics (Caucasian, age, dorsal skin), to verify reflection and absorption bands and it shows very good overlapping (not shown) underlying the reproducibility of the system.

Spectral shape obtained also confirmed what already found by Sun and co-workers (Sun et al. 2024), thus the measured spectral reflectance has a V shape between 400 and 450 nm, and a W shape between 520 and 610 nm, (found in all the ethnic groups, including the Caucasian, object of this study) suggesting the relevance of these wavelengths ranges in describing hand skin healthy pattern profile.

Seasonal variation could be considered as source of possible confounders, while inducing skin modifications. For what concern the possible impact due to tanning, all the subjects who participated in the study were recruited between June 2024 and March 2025, but avoiding the months of July, August, and September, mainly due to closures during the summer holidays. Therefore, we rule out the possibility that tanning could have a significant impact on the subjects of this cohort. Regarding skin hydration and its modification, we do not have a direct measure of them, and this is a limitation of our work. Despite this, we have extracted a measure of water content from hyperspectral images (the spectral parameter TWI*) and, since it does not show correlation with seasonal variation, we used it in the analysis. Future studies involving metabolomics on the same subjects will allow to complement this task with the evaluation of cutaneous metabolites that could be related to tissue hydration.

Features selection (see Table 3) has highlighted a group of descriptors obtained from spectral shape and radiomics. $\textrm{Flex}_{Palm}$, $\textrm{TWI}^{*}_{Back}$ and $\textrm{NDI}_{Back}$ belong to the first group. Considering mean spectral parameters, $\textrm{TWI}^{*}_{Back}$ demonstrates a declining trend consistent with the aging process (Li et al. 2023) with a supposed more pronounced pattern observed in the female subset, here not observed (Supplemental Fig. S5) probably because of not sufficient size of data set or due to the operation range and resolution of Specim IQ camera. $\textrm{NDI}_{Back}$" increases with a similar trend but not $\textrm{Flex}_{Palm}$ that behaves different while comparing male and female (Supplemental Fig. S5), however it is difficult to draw conclusions about this difference, given the narrow range of values observed.

Considering GLCM parameters (Fig. 3), Contrast value (observed at 470 nm in Palm, 565 nm in Back, 575 nm in Back) increases with age, while Joint Energy (470 nm in Back, 565 nm in Back, 575 nm in Back), decreases, suggesting an inverse relationship. The reiterate presence of Contrast and Joint Energy among the selected significant variables, found for three of the four wavelengths (470, 565, 575 nm) used for the radiomic approach, confirms their relevance in determining the healthy profile and depicting the process of progressive loss of tissue integrity. GLCM Contrast measures the overall gray-level dynamic range, so its increase can suggest less uniformity (Ardakani et al. 2021) in hand tissue texture. On the contrary, a decrease in GLCM Joint Energy indicates that the texture loses its uniform or regular pattern. Also, the inverse relationships between contrast and Idm is confirmed by literature and, similarly to Joint Energy, it implies a decreasing homogeneity while aging increases.

These findings are confirmed by Difference Entropy (540 nm in Back), a measure of the randomness/variability in neighborhood intensity value differences, that increases with aging. Finally, Maximum Probability (540 nm in Back), which quantifies the occurrence of the most predominant pair of neighboring intensity values, decreases with age, highlighting once again the loss of skin homogeneity.

Taken together, these features underline that the aging process in healthy skin condition is mainly characterized by an increased pattern of heterogeneity and a progressive decline in water content, suggesting them as promising variables to be monitored. Furthermore, several of these descriptors (water content, oxygenation-related indices and texture parameters) can be interpreted as indirect markers of skin metabolic status, as they reflect changes in microvascular perfusion, oxygen delivery, extracellular matrix turnover and tissue hydration along aging. In the end, Spline curves obtained by the analysis suggest the accepted range of them in healthy individuals. Wearable devices for skin health monitoring could be easily implemented taking into account these results.

Previous works have already studied human dorsal hand skin texture for assessing the skin aging process, among them Durai and co-workers (Durai et al. 2012), and Lu and co-workers (Lu et al. 2025a), however they are not specifically oriented to find a group of descriptors able to define the range of the healthy skin status while aging.

The work by Durai and co-workers collects a huge number of samples of skins from different sites (hair, nails, palms, soles, genitalia and teeth) but it is a descriptive and qualitative evaluation, based on operator-dependent dermatological examination. The International Skin Spectra Archive (ISSA) by Lu and co-workers creates an exhaustive archive for the evaluation of the skin colour in different human phenotypes in the visible range (400 - 700 nm). We used this dataset as a reference for validating our measurements, particularly for notable points identified in Fig. 2. However, the repository mainly serves as a data resource and does not include analyses or interpretations of the reported spectra, leaving these aspects open for discussion. Similarly to our study, Calin and co-workers (Calin et al. 2016) focus on texture analysis to assess the skin aging process. However, their results basically rely on GLCM features characterization, potentially lacking information carried out by the spectral profile. Moreover, they suggest HSI as reliable tool for skin aging study but they don’t point out an effective group of parameters for aging monitoring.

According to the protocol of the project, other data have been collected from the healthy volunteers and, in detail, epigenetic data (miRNAs), metabolomic data and grip strength. Further analysis will be performed by integrating multimodal data for characterizing aging process. In this framework, the present panel of HSI-derived parameters may represent a first step toward defining an optical signature of skin metabolic health during physiological aging, opening to non-invasive monitoring of tissue-level metabolic alterations. This integrated approach may also strengthen the interpretation of these optical parameters as surrogate markers of metabolic and genetic pathways involved in healthy aging.

A limitation of the study is represented by a still limited number of volunteers in particular considering subject with age ≥ 80 and by the restricted area of provenance of subjects which limits the variability of exposome. In addition, widening range of wavelenghts can improve the analysis by the evaluation of additional parameters, particularly in the mid- and far-infrared regions of the electromagnetic spectrum.

Overall, these results provide a comprehensive framework for using hyperspectral imaging to non-invasively characterize and monitor skin health across aging processes, laying the groundwork for personalized and longitudinal assessments of healthy aging processes.

## Supplementary Information

Below is the link to the electronic supplementary material.

Supplementary file 1 (pdf 8464 KB)

## Data Availability

All data supporting the findings of this study are available under request to the corresponding author.
