# Supplementary material for: Hyperspectral imaging and healthy aging: an observational study using hand skin as surface for monitoring healthy aging processes
Source: Biogerontology. 2026 Jun 17;27(4):116. doi: 10.1007/s10522-026-10461-w (PMC13275750; doi:10.1007/s10522-026-10461-w)
Supplement: Supplementary file 1 — (pdf 8464 KB) [file 10522_2026_10461_MOESM1_ESM.pdf]

Supplemental Material

Questionnaire on Lifestyle and Well-being in Active Aging

Introduction

This survey investigates lifestyle and self-perceived well-being in active aging, within the project *"Integrated Innovative Technologies for Developing a One-Health Approach in the Elderly Population"*. People over 40 years represent an increasing portion of the population across Europe. The *OneHealth* approach integrates multiple factors influencing healthy aging, including lifestyle.

This questionnaire is voluntary and anonymous. Data are treated confidentially.

Questions are adapted from the SHARE project: `https://share-eric.eu/data/data-documentation/questionnaires`.

Fields marked with * are mandatory. Entry ID code to be filled in by the principal researcher.

Background Information

Entry code* ____________________

Age* ____________________

Biological sex*
☐ Male ☐ Female

Nationality* ____________________

Education*
☐ Elementary ☐ Middle ☐ High School ☐ Degree ☐ PhD

Employment status
☐ Employed ☐ Retired ☐ Unemployed

Household size*
☐ 0 ☐ 1 ☐ 2 ☐ 3 ☐ > 3

Housing type*
☐ Flat ☐ Detached ☐ Other

Do you take medications regularly?*
☐ Yes ☐ No

If yes, specify: ____________________

Self-perceived Well-being

Health status*
☐ Very good ☐ Good ☐ Fair ☐ Poor ☐ Very poor

Impact of work on quality of life*
☐ Very positive ☐ Positive ☐ Neutral ☐ Negative ☐ Very negative

Negative factors affecting daily life*
☐ Work pressure ☐ Social relations ☐ Family relations ☐ Daily stress
☐ Commuting ☐ Physical work ☐ Job stability ☐ Loneliness ☐ None

Environment and Lifestyle

Green areas availability*
☐ Abundant ☐ Adequate ☐ Insufficient ☐ None

Pollution level*
☐ High ☐ Moderate ☐ Low ☐ None

Main pollution source*
☐ Traffic ☐ Industry ☐ Landfills ☐ Farming ☐ None

Habits

Ever smoked regularly (1 year)*
☐ Yes ☐ No

Currently smoking*
☐ Yes ☐ No

Alcohol consumption (last 3 months)*
☐ Daily ☐ 5–6 days/week ☐ 3–4 days/week ☐ 1–2 days/week ☐ Monthly
☐ Never

Dairy consumption*
☐ Daily ☐ 3–6/week ☐ 2/week ☐ 1/week ☐ Rarely ☐ Never

Legumes / eggs*
☐ Daily ☐ 3–6/week ☐ 2/week ☐ 1/week ☐ Rarely ☐ Never

Meat / fish*
☐ Daily ☐ 3–6/week ☐ 2/week ☐ 1/week ☐ Rarely ☐ Never

Fruit / vegetables*
☐ Daily ☐ 3–6/week ☐ 2/week ☐ 1/week ☐ Rarely ☐ Never

Vigorous physical activity*
☐ $>$ 1/week ☐ 1/week ☐ 1–3/month ☐ Never

Moderate physical activity*
☐ $>$ 1/week ☐ 1/week ☐ 1–3/month ☐ Never

Questionnaire item score

Participants completed a questionnaire, allowing to obtain socio-demographic data as well as information about participants' lifestyle. Questionnaire items were suggested and adapted by the analysis of surveys proposed in SHARE research infrastructure `https://share-eric.eu/data/data-access/citation-requirements`. The choice of items proposed to the participants was guided, on one hand, by the need to have sufficient information to be able to define key aspects of the participants' exposome. On the other hand, the number of questions was limited to avoid making the questionnaire itself too complex and long to complete.

We adopted an a priori, top-down approach to define the thematic dimensions of interest as commonly used in social sciences, public health, and lifestyle research (Bhattacherjee, 2019; Krägeloh et al., 2023). In particular, we defined a set of four coherent and conceptually meaningful domains which can be considered as impacting in defining the healthy aging (World Health Organization, 2024): Protective Lifestyle Behaviors (Hendriks, 2020; Hu et al., 2018; Minzer et al., 2020; Plassman, 2010; Russo et al., 2022; Tessier et al., 2025; Turrini et al., 2023; Zhang, 2023), Perceived Well-Being (García-Muñoz et al., 2018; Siegrist et al., 2009; Trică et al., 2024), Educational Level (Hernandez et al., 2025) and Social Engagement (Siegrist and Wahrendorf, 2016). Protective Lifestyle Behaviors domain includes items concerning eating habits, alcohol consumption, smoking, and physical activity. Perceived Well-Being domain includes items concerning the perception related to one's own health status, the impact of work, and contributing factors such as, for example, social pressure or the family environment. Social Engagement domain is a measure of social interaction while Educational Level measures the education level of participants.

Questionnaire items associated to each domain were then scored on the basis of literature evidence considering the impact that each item has to the respective assigned domain. For example, considering that intense physical activity is associated with evident benefits for healthy aging, engaging in intense physical activity results in an increase in the score of Protective Lifestyle Behaviors.

Once all the items scores have been evaluated and normalized, the score of each domain was computed by summing all the items scores belonging to each domain.

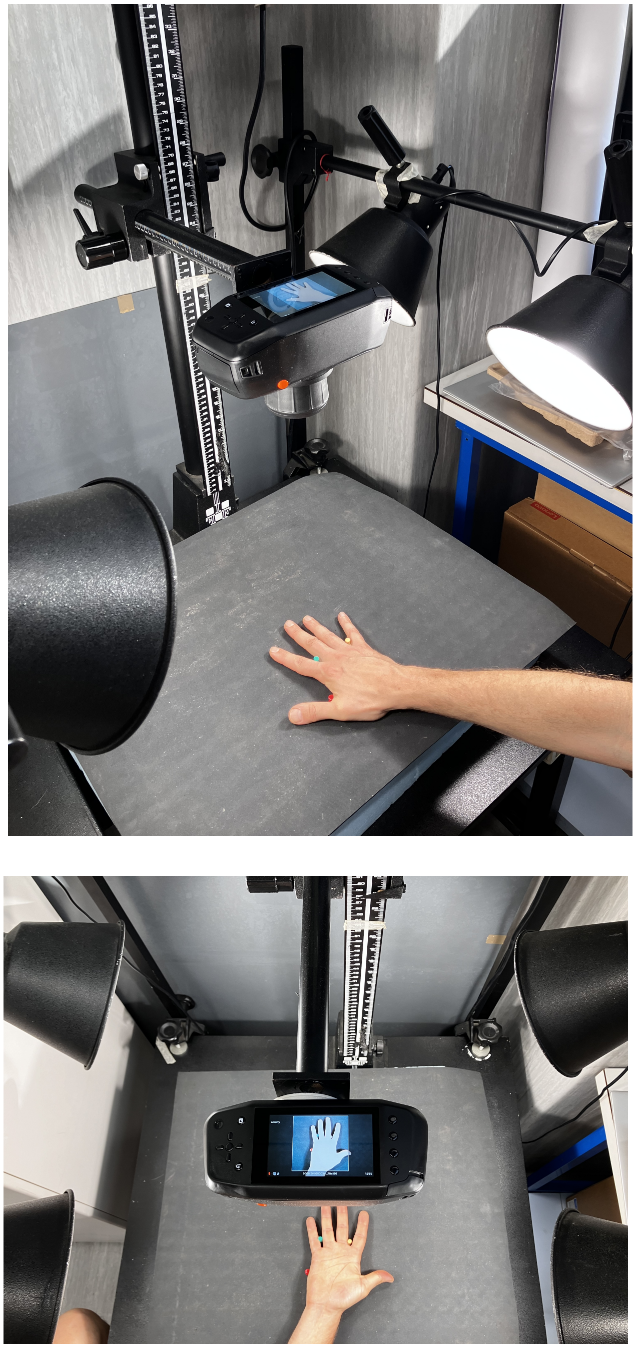

Figure S1: Acquisition setting: the Specim IQ camera is place on the top of the stand and four led sources (19 W - 245 lm) are placed closer, at the same distance, 45° oriented, to ensure illumination uniformity toward the hand. Pins are fixed to table to allow the right positioning of the hand and the reproducibility of the acquisitions.

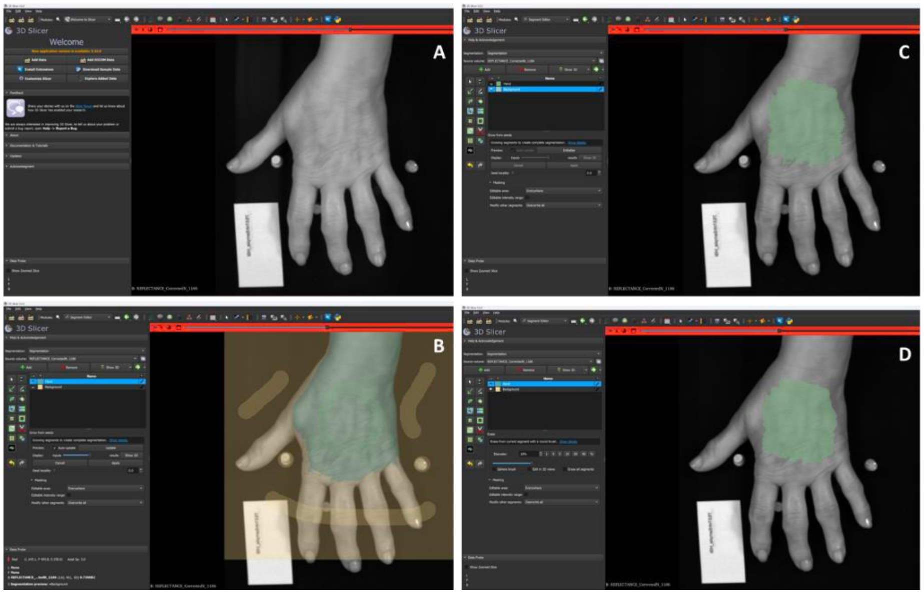

Figure S2: 3D slicer segmentation process 1) The image to be segmented 2) The user defines the two regions (hand and background) and draws the seeds. 3) The user adjusts the results of the segmentation process according to her/his needs. 4) The edges of the ROI are smoothed

Spectral parameters

As described in the main text the sigmoidal parameters have been obtained by fitting a sigmoidal growth model to the mean spectra in the 575 - 670 nm range. Figure S3 shows an example where the meaning of each parameter has been recalled by green texts. In the formulas describing Literature and Shape parameters in the main text (Table 1 in the main text), the subscript of R indicates the wavelength corresponding to the reflectance values used for evaluating the parameters. The angle brackets $\langle .... \rangle$ indicate that has been used the average value of the reflectance in the range specified by the subscripts.
The parameters THI*, TWI* and NIRind* have been adapted from Pachyn and co-workers (Pachyn et al., 2024). The superscript * highlights that these parameters are not exactly the same as those used in the cited work. In facts, while Pachyn and co-workers extract the indices form absorbance measurements, here we exploit reflectance measurements; this has been done under the infinitely tick sample approximation, where the portion of light transmitted through the sample can be neglected and the reflectance considered as inversely proportional to the absorbance. In the formulas for THI*, TWI* and NIRind*, s_1 and s_2 are constant that do not affect the parameters distribution and can be neglected as done by Pachyn and co-workers (Pachyn et al., 2024).

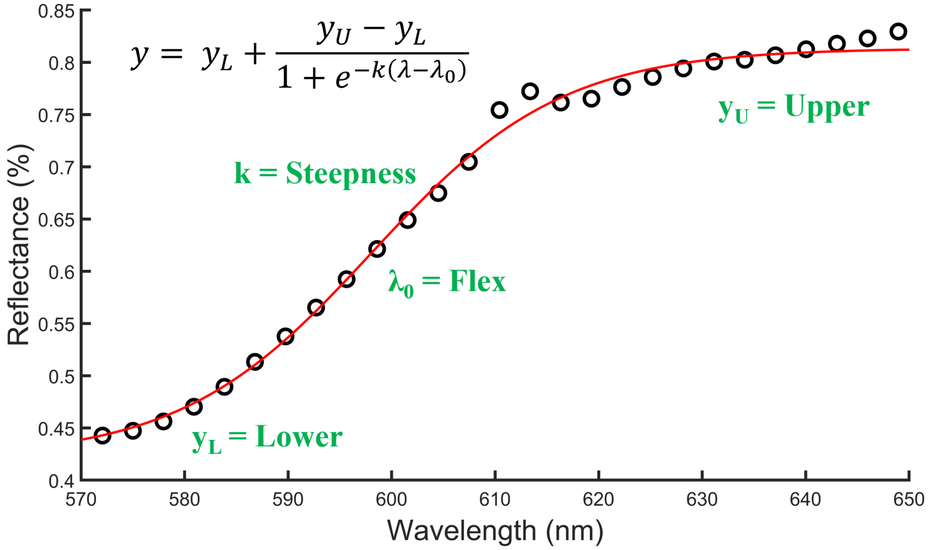

Figure S3: Example of sigmoidal model (red line) fitting the mean reflectance spectrum (dots); green labels indicate Sigmoidal Parameters used in the analysis.

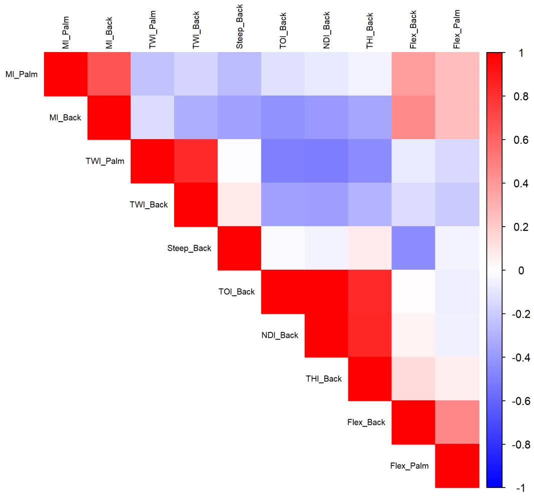

(a) Spectral Features

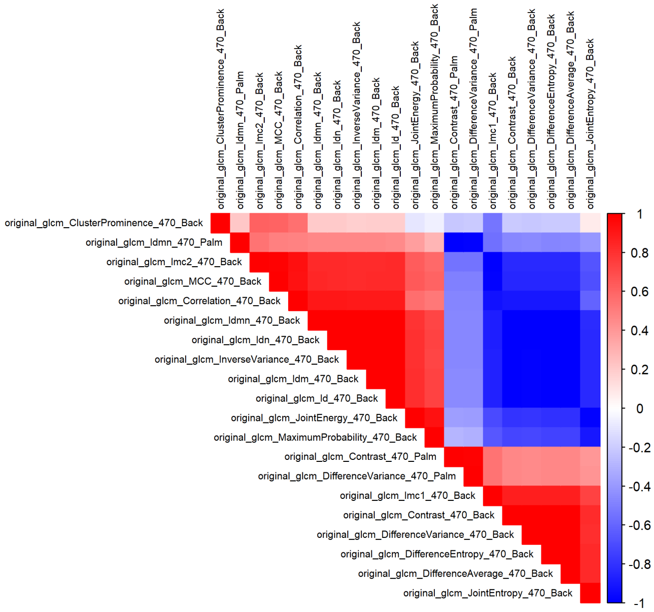

(b) 470 nm Radiomic Features

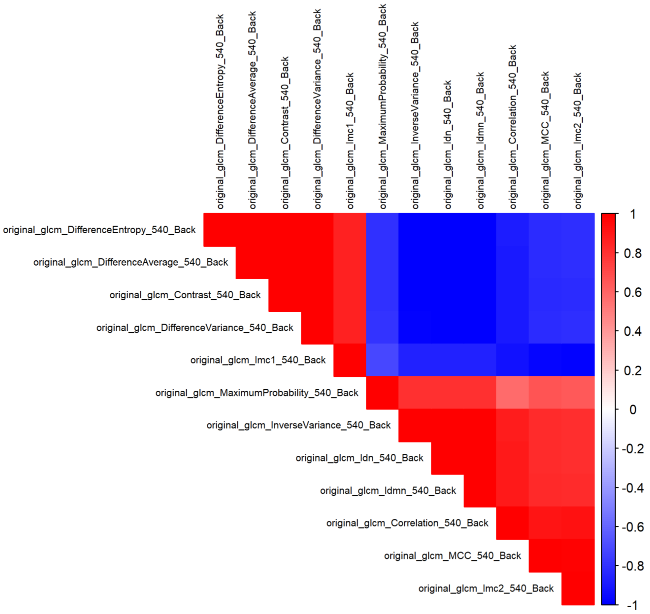

(c) 540 nm Radiomic Features

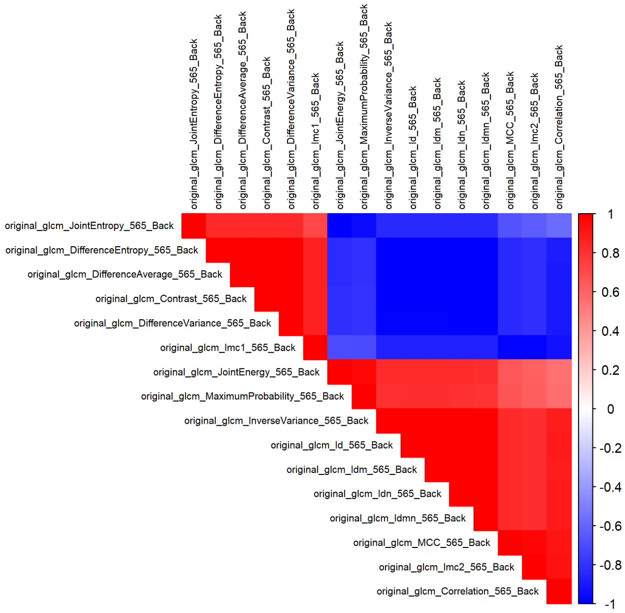

(d) 565 nm Radiomic Features

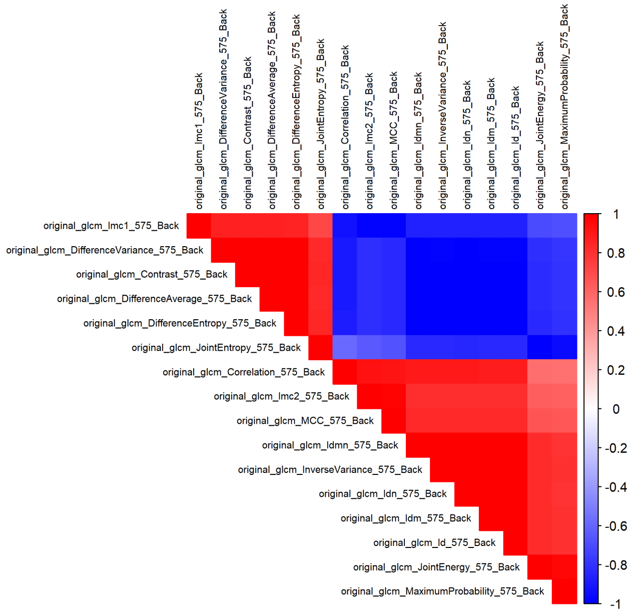

(e) 575 nm Radiomic Features

Figure S4: Correlation Matrices showing features redundancies according to their referenced group. Panels represent features from: (a) Spectral data, (b) GLCM on 470 nm, (c) GLCM on 540 nm, (d) GLCM on 565 nm, (e) GLCM on 575 nm.

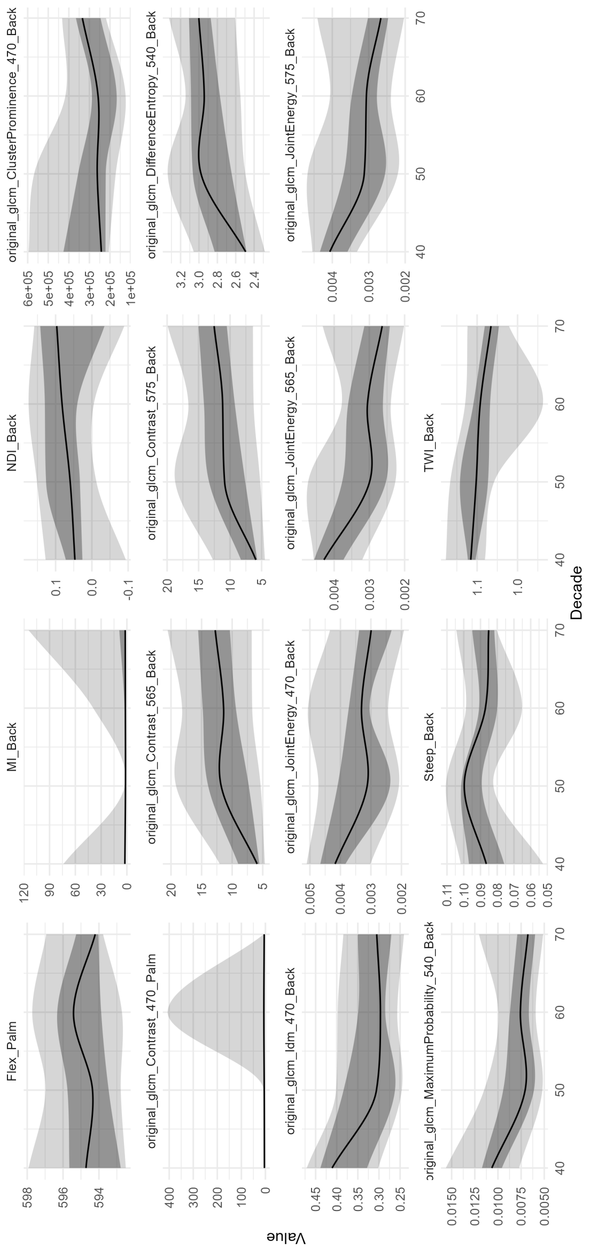

(a) Female

Figure S5: Reference curves of "healthy status" for the 15 parameters retained after feature selection plotted according to decades and sex (part 1).

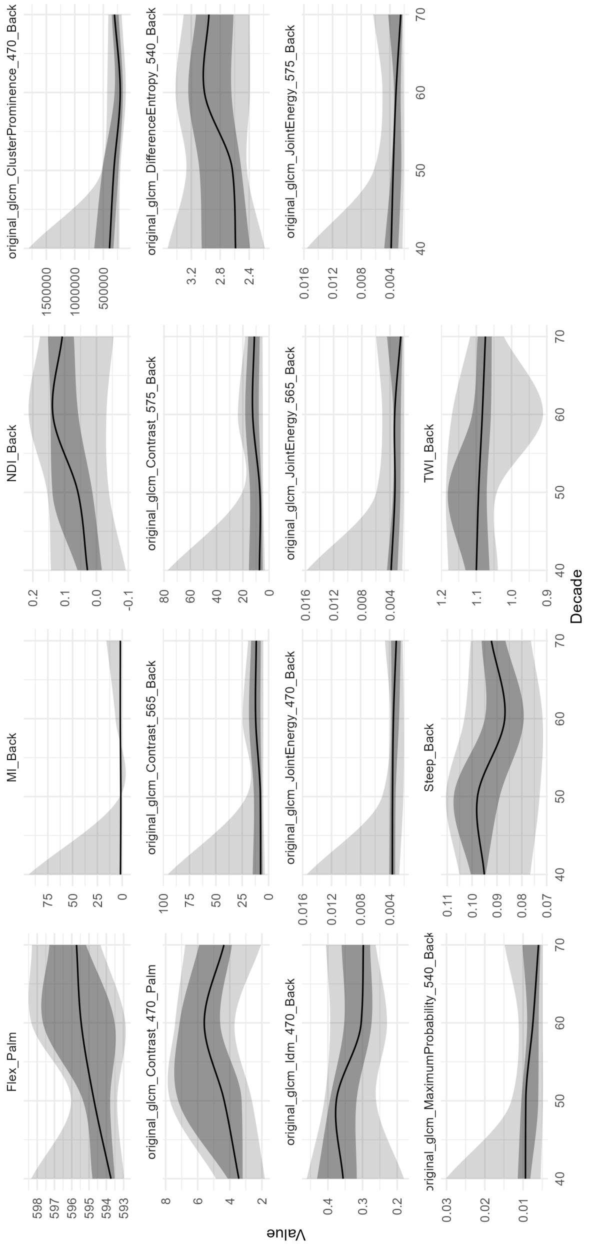

(b) Male

Figure S5: Reference curves of "healthy status" for the 15 parameters retained after feature selection plotted according to decades and sex (part II).
Each curve is modeled using a spline function and corresponds to a single parameter. Outer light grey area corresponds to 5th–95th percentile range, inner dark grey area to 25th–75th percentile, line to median (50th percentile).
(a) corresponds to female subgroup, (b) corresponds to male subgroup.

References

Bhattacherjee, A. (2019). *Social Science Research: Principles, Methods and Practices.* University of Southern Queensland, revised edition edition.

García-Muñoz, T., Neuman, S., and Neuman, T. (2018). Behavioral health risk factors: the interaction of personal and country effects. *International Journal of Behavioral Medicine*, 25(2):183–197.

Hendriks, H. F. J. (2020). Alcohol and human health: What is the evidence? *Annual Review of Food Science and Technology*, 11:1–21.

Hernandez, H., Santamaria-Garcia, H., Moguilner, S., et al. (2025). The exposome of healthy and accelerated aging across 40 countries. *Nature Medicine*, 31:3089–3100.

Hu, Y., Zong, G., Liu, G., Wang, M., Rosner, B., Pan, A., Willett, W. C., Manson, J. E., Hu, F. B., and Sun, Q. (2018). Smoking cessation, weight change, type 2 diabetes, and mortality. *New England Journal of Medicine*, 379(7):623–632.

Krägeloh, C. U., Alyami, M., and Medvedev, O. N., editors (2023). *International Handbook of Behavioral Health Assessment.* Springer, Cham, Switzerland.

Minzer, S., Losno, R. A., and Casas, R. (2020). The effect of alcohol on cardiovascular risk factors: Is there new information? *Nutrients*, 12.

Pachyn, E., Aumiller, M., Freymüller, C., Linek, M., Volgger, V., Buchner, A., Rühm, A., and Sroka, R. (2024). Investigation on the influence of the skin tone on hyperspectral imaging for free flap surgery. *Scientific Reports*, 14(1):13979.

Plassman, B. L. (2010). Factors associated with risk for and possible prevention of cognitive decline in later life. *Annals of Internal Medicine*, 153.

Russo, C., Walicka, M., Caponnetto, P., Cibella, F., Maglia, M., Alamo, A., Campagna, D., Frittitta, L., Di Mauro, M., Caci, G., Krysinski, A., Franek, E., and Polosa, R. (2022). Efficacy and safety of varenicline for smoking cessation in patients with type 2 diabetes: A randomized clinical trial. *JAMA Network Open*, 5(6):e2217709.

Siegrist, J. and Wahrendorf, M. (2016). Failed social reciprocity beyond the work role. In Siegrist, J. and Wahrendorf, M., editors, *Work Stress and Health in a Globalized Economy: The Model of Effort-Reward Imbalance*, pages 275–291. Springer International Publishing, Cham.

Siegrist, J., Wege, N., Pühlhofer, F., and Wahrendorf, M. (2009). A short generic measure of work stress in the era of globalization: effort-reward imbalance. *International Archives of Occupational and Environmental Health*, 82(8):1005–1013.

Tessier, A. J. et al. (2025). Optimal dietary patterns for healthy aging. *Nature Medicine*, 31(5):1644–1652.

Trică, A., Golu, F., Sava, N. I., Licu, M., Zanfirescu, A., Adam, R., and David, I. (2024). Resilience and successful aging: A systematic review and meta-analysis. *Acta Psychologica*, 248:104357.

Turrini, S., Wong, B., Eldaief, M., Press, D. Z., Sinclair, D. A., Koch, G., Avenanti, A., and Santarnecchi, E. (2023). The multifactorial nature of healthy brain ageing: Brain changes, functional decline and protective factors. *Ageing Research Reviews*, 88:101939.

World Health Organization (2024). *Measuring the progress and impact of the UN Decade of Healthy Ageing (2021–2030).* World Health Organization.

Zhang, Y. (2023). Healthy aging and public health perspectives. *Frontiers in Public Health.*
